# Gastrointestinal neuroendocrine tumor with discordant metastatic disease on 111In-pentetreotide SPECT/CT, 18F-DOPA PET/CT and 68Ga-HA-DOTATATE PET/CT

**DOI:** 10.1186/s41824-022-00134-5

**Published:** 2022-07-04

**Authors:** Katrin Resch, Ryan Hung, Jonathan Abele

**Affiliations:** Department of Radiology and Diagnostic Imaging, 2A2.41 WC Mackenzie Health Sciences Centre, University of Alberta, 8440 112 Street NW, Edmonton, AB T6G 2B7 Canada

**Keywords:** Neuroendocrine tumor, Fluorodopa F 18, 68Ga-HA-DOTATATE, 177Lu-DOTATATE

## Abstract

A 62-year-old man with resected, pathology-proven small bowel neuroendocrine tumor underwent 111In-pentetreotide SPECT/CT, 18F-DOPA PET/CT and 68Ga-HA-DOTATATE PET/CT to assess metastatic disease. The 111In-pentetreotide SPECT/CT scan showed no metastatic disease. Both 18F-DOPA and 68Ga-HA-DOTATATE PET/CT showed hepatic and peritoneal metastatic disease. However, the burden of 18F-DOPA-avid metastatic disease was far greater compared to the burden of 68Ga-HA-DOTATATE-avid metastatic disease.

## Introduction

Many nuclear medicine tracers are available for NET detection and surveillance. A distinctive feature of NET cells is that most of them (70–90%) overexpress somatostatin receptors (Johnbeck et al. 2014). This has allowed somatostatin receptors to be used as targets for molecular imaging. Traditionally, the most commonly used radiotracer to identify somatostatin receptor-positive tumors has been 111In-pentetreotide, which is a single-photon emission computed tomography (SPECT) agent.

However, positron emission tomography (PET)-based isotopes for somatostatin receptor imaging have been developed and are increasingly utilized. PET provides higher spatial resolution images than SPECT. 68Ga-DOTATATE PET has been shown to have higher sensitivity and specificity for NETs than 111In-pentetreotide SPECT (Mojtahedi et al. 2014). The reported sensitivity of 68Ga-DOTA-peptide PET/CT imaging for NETs is between 88 and 93%, and the specificity is between 88 and 95% (Sundin 2018).

18F-DOPA is another PET agent that has high sensitivity and specificity for NETs. 18F-DOPA has a different mechanism of action than somatostatin analogues. 18F‐FDOPA is taken up into NET cells via an amino acid transporter (LAT1/CD98). The 18F-DOPA is then decarboxylated within the cell and becomes trapped intracellularly. NETs over-express the LAT1/CD98 amino acid transport system, which leads to increased 18F-DOPA uptake by NETs (Santhanam and Taïeb 2014). A meta-analysis by Rufini et al. found an overall sensitivity of 77% and specificity of 95% for the diagnosis of NETs with 18F-DOPA, with the sensitivity varying depending on the location of the NET (Rufini et al. 2013). Given the alignment with 177Lu-DOTATATE therapy, 68Ga-DOTATATE has become increasingly prevalent for NET diagnosis and staging (Mittra 2018; Strosberg et al. 2017).

## Case report

A 62-year-old man presented with nausea, vomiting, altered bowel habits, weight loss, fevers and night sweats. At presentation, he initially had an IV contrast-enhanced CT examination of the abdomen and pelvis. This revealed a small bowel obstruction caused by an inflammatory mass at the terminal ileum. The patient underwent an open right hemicolectomy with resection of the mass at the ileocecal junction. The surgeon also noted tethering of the mesentery and omentum stuck down in this area. The surgeon also visualized large nodes in the small bowel mesentery, a couple of small peritoneal plaques in the pelvis, and minor studding along the falciform ligament. There were also plaques on both hemidiaphragms.

Pathology showed a well-differentiated 3.2-cm neuroendocrine tumor, grade 2, with lymphovascular and perineural invasion present. Five out of 21 nodes were positive. The sampled falciform ligament was also positive for metastatic well-differentiated neuroendocrine tumor. Thus, he had a T3N2M1b neuroendocrine tumor of the small bowel.

**Fig. 1:**
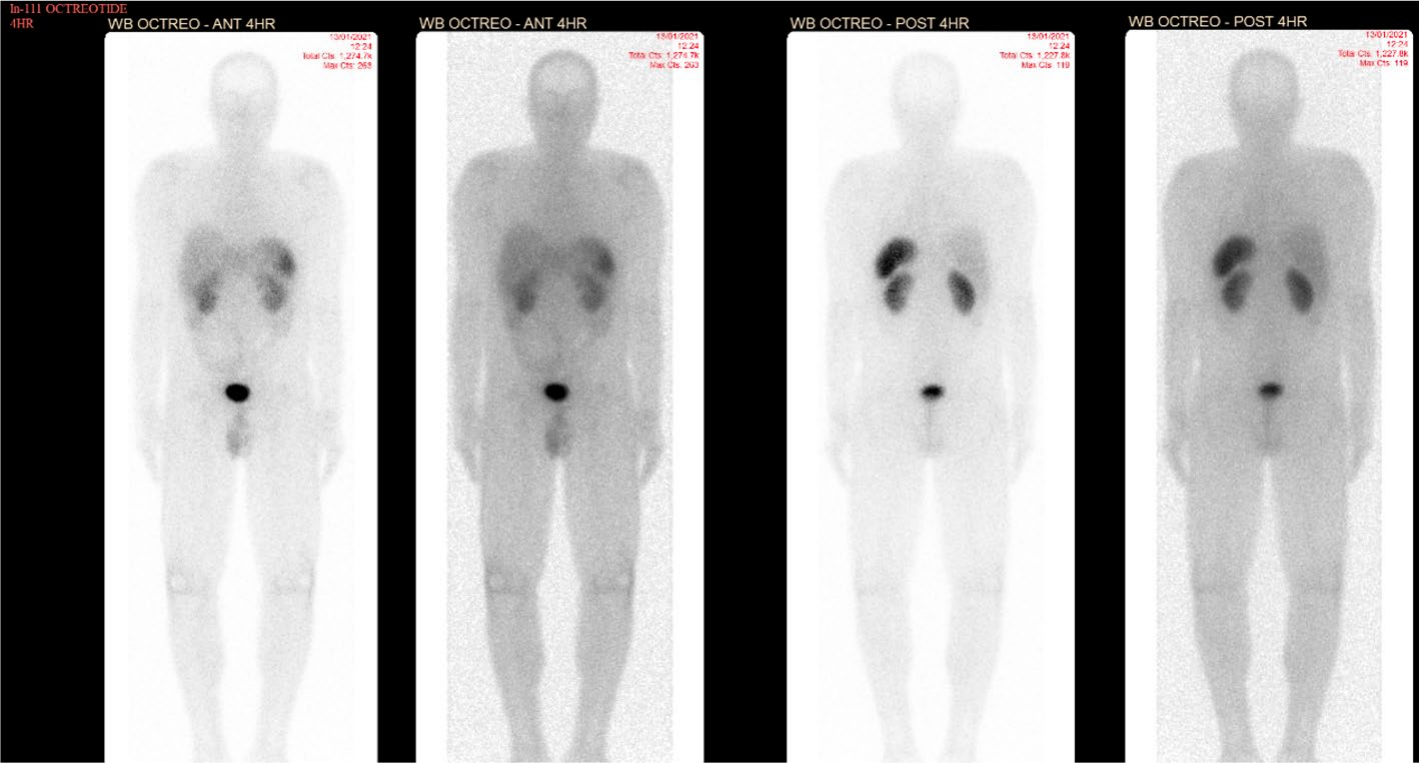
Planar anterior and posterior whole-body 111In-pentetreotide imaging acquired at 4 h and 24 h demonstrating no pentetreotide-avid disease

Approximately 1.5 months postoperatively, the patient had an enhanced CT abdomen and pelvis to look for recurrent and/or metastatic disease. The CT scan showed an enhancing liver nodule. There were also several enlarged right external iliac chain lymph nodes and lymph nodes in the right hemipelvis. However, the 111In-pentetreotide SPECT/CT scan performed 3 days later was negative for any pentetreotide-avid disease (Fig. 1). No activity was seen associated with the enlarged lymph nodes or the liver lesion. This was the first nuclear medicine study performed, as 111In-pentetreotide is currently still the most available neuroendocrine tracer at our institution.

**Fig. 2:**
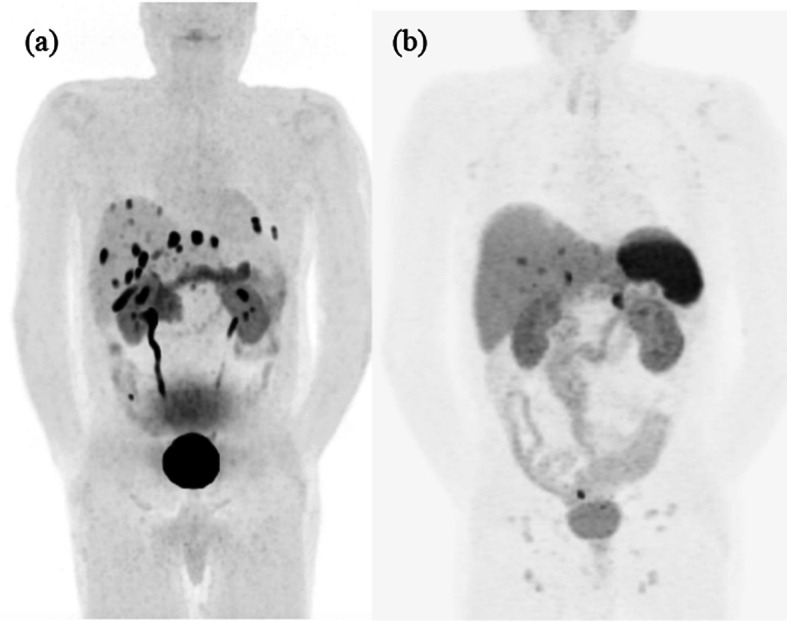
18F-DOPA PET/CT MIP (**a**) and 68Ga-HA-DOTATATE PET/CT MIP (**b**) performed 22 days apart. While some metastases were DOTATATE-avid, the 18F-DOPA scan revealed many more sites of discordant non-DOTATATE-avid disease

**Fig. 3:**
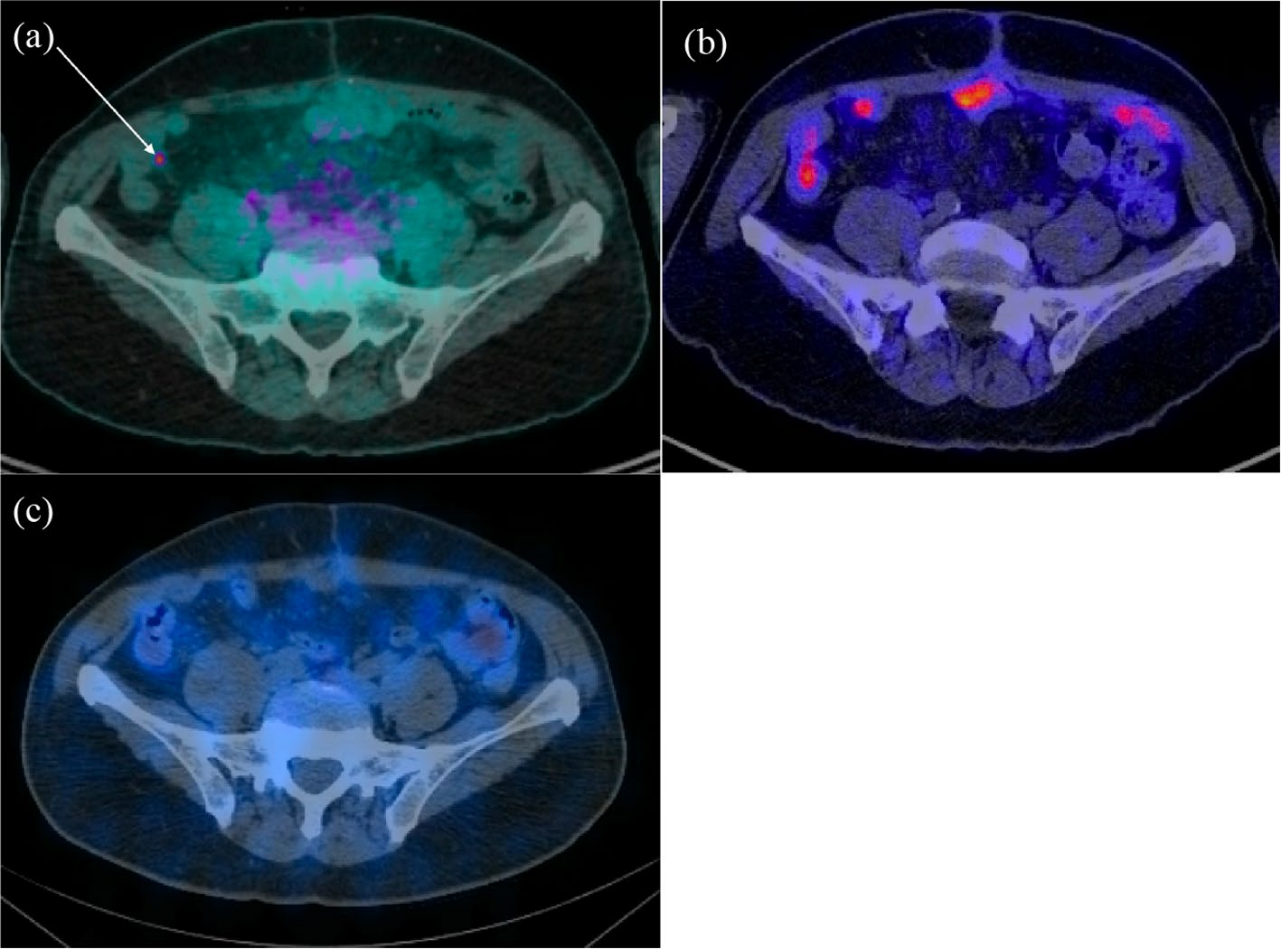
18F-DOPA PET/CT (**a**), 68Ga-HA-DOTATATE PET/CT (**b**) and 111In-pentetreotide SPECT/CT (**c**) axial images of the pelvis. Intensely avid 0.3-cm lymph node within the right lower quadrant mesentery adjacent to the neoterminal ileum demonstrated intense 18F-DOPA activity (**a**), but no 68Ga-HA-DOTATATE (**b**) or 111In-pentetreotide (**c**) avidity

An 18F-DOPA PET/CT scan (Fig. 2) using 322 MBq of 18F-DOPA with a 1-h uptake period was performed 27 days after the 111In-pentetreotide SPECT/CT scan. An intensely 18F-DOPA-avid right mesenteric lymph node adjacent to the neoterminal ileum was identified, in keeping with a nodal metastasis (Fig. 3). There were also intensely 18F-DOPA-avid peritoneal deposits within the pelvis and in the left upper quadrant. Multiple 18F-DOPA-avid hepatic lesions were also identified, also consistent with metastatic disease.

Only 22 days after the 18F-DOPA PET/CT, a 68Ga-HA-DOTATATE PET/CT was performed with 230 MBq of 68Ga-HA-DOTATATE (Fig. 2). The patient had not yet started therapy. The DOTATATE study was performed to see if the patient would benefit from 177Lu-DOTATATE therapy. The 18F-DOPA-avid right mesenteric lymph node demonstrated no DOTATATE avidity (Fig. 3). Several hepatic lesions were again identified, but hepatic disease burden was significantly underestimated compared to the 18F-DOPA study. Also, fewer peritoneal metastatic deposits were identified on the DOTATATE study.

## Conclusion

Both 18F-DOPA and 68Ga-DOTATATE have high sensitivity and specificity for NETs, with increased lesion detection by 18F-DOPA (Piccardo et al. 2021). These tracers are useful to identify the extent of neuroendocrine tumor burden, as demonstrated in this patient with a negative 111In-pentetreotide SPECT/CT scan, but evidence of metastatic NETs on 18F-DOPA PET/CT and 68Ga-HA-DOTATE PET/CT. Furthermore, some lesions are avid for both PET tracers and some only for one in the same patient. Given the alignment with therapy, 68Ga-HA-DOTATATE has become increasingly prevalent for NET diagnosis and staging. However, as demonstrated in this case, 18F-DOPA can identify additional discordant (non-DOTATATE-avid) lesions, which has important implications in terms of both treatment selection and prognosis.

While the potential significance of discordance between DOTATATE and 18F-FDG has been acknowledged (You et al. 2020), the clinical impact of DOTATATE/DOPA discordant disease is unknown and requires further investigation, particularly in light of planning personalized therapies such as 177Lu-DOTATATE.

## Data Availability

Not applicable.
